# Microarray meta-analysis reveals IL6 and p38β/MAPK11 as potential targets of hsa-miR-124 in endothelial progenitor cells: Implications for stent re-endothelization in diabetic patients

**DOI:** 10.3389/fcvm.2022.964721

**Published:** 2022-09-13

**Authors:** Alberto Arencibia, Luis A. Salazar

**Affiliations:** Department of Basic Sciences, Faculty of Medicine, Center of Molecular Biology and Pharmacogenetics, Universidad de La Frontera, Temuco, Chile

**Keywords:** circulating progenitor endothelial cells, stent restenosis, diabetes, microarray meta-analysis, micro-RNA

## Abstract

Circulating endothelial progenitor cells (EPCs) play an important role in the repair processes of damaged vessels, favoring re-endothelization of stented vessels to minimize restenosis. EPCs number and function is diminished in patients with type 2 diabetes, a known risk factor for restenosis. Considering the impact of EPCs in vascular injury repair, we conducted a meta-analysis of microarray to assess the transcriptomic profile and determine target genes during the differentiation process of EPCs into mature ECs. Five microarray datasets, including 13 EPC and 12 EC samples were analyzed, using the online tool ExpressAnalyst. Differentially expressed genes (DEGs) analysis was done by Limma method, with an | log_2_FC| > 1 and FDR < 0.05. Combined *p*-value by Fisher exact method was computed for the intersection of datasets. There were 3,267 DEGs, 1,539 up-regulated and 1,728 down-regulated in EPCs, with 407 common DEGs in at least four datasets. Kyoto Encyclopedia of Genes and Genomes (KEGG) analysis showed enrichment for terms related to “AGE-RAGE signaling pathway in diabetic complications.” Intersection of common DEGs, KEGG pathways genes and genes in protein-protein interaction network (PPI) identified four key genes, two up-regulated (IL1B and STAT5A) and two down-regulated (IL6 and MAPK11). MicroRNA enrichment analysis of common DEGs depicted five hub microRNA targeting 175 DEGs, including STAT5A, IL6 and MAPK11, with hsa-miR-124 as common regulator. This group of genes and microRNAs could serve as biomarkers of EPCs differentiation during coronary stenting as well as potential therapeutic targets to improve stent re-endothelization, especially in diabetic patients.

## Introduction

Circulating endothelial progenitor cells (EPCs) are a heterogeneous group of circulatory cells that play an important role in the repair processes in damaged tissues (1). EPCs are mobilized from the bone marrow into the circulation in response to tissue damage (2) and have the capacity to home to injured blood vessels and differentiate in mature endothelial cells (ECs) (3).

In the context of acute coronary syndrome treated with percutaneous coronary intervention (PCI) there is a profound damage of the vascular wall (4). Coronary stenting results in clinical or subclinical neointima formation with stent restenosis in about 30% of patients (5). Coronary stenting cause a burst of EPCs within 24 h when comparing to balloon angioplasty (6). Drug eluting stents (DES) avoid neointima formation by vascular smooth muscle cells, but also target ECs, limiting stent re-endothelization and favoring in-stent restenosis and thrombosis (7).

EPCs have been extensively investigated for their capacity to modulate neointimal formation in PCI (8). Recruiting EPCs into the stent with CD34 + (9) or CD133 + (10) antibodies promote early re-endothelization while reducing the risk of stent thrombosis and in-stent restenosis (11) when comparing with bare metal stents (BMS) (12).

Reduction of EPCs has been proposed as a novel mechanism of cardiovascular disease in type 2 diabetes. In comparison with normoglycemic patients, diabetic patients have a significant reduction of EPCs number and function with increased apoptosis (13). Another investigation described impaired number, migration, CXCR4 expression, and nitric oxide (NO) production in EPCs from diabetes patients and were further reduced in patients with coexisting coronary artery disease. The expression of CXCR4 and activation of Pi3K/Akt/eNOS signaling cascade were suppressed in cultured EPCs treated with hyperglycemia and oxidized LDL (14).

A recent meta-analysis showed that lower baseline EPCs count has been associated with a significantly greater occurrence of in-stent restenosis (HR 1.33; 95% CI 0.97–1.82, *P* = 0.045). Nevertheless, in EPCs-capturing DES, target lesion revascularization was significantly more common than with standard DES (15).

Considering the theoretical and practical impact of EPCs in vascular injury repair, we conducted a meta-analysis of microarray to assess the transcriptomic profile and determine targets genes during the differentiation process of EPC into mature EC.

## Materials and methods

### Data compilation and processing

We queried the Gene Expression Omnibus (GEO) repository using the following terms: “endothelial progenitor” [All Fields] AND “Homo sapiens” [porgn: txid9606] AND “Expression profiling by array” [Filter]. Datasets were manually curated to select those studies comparing EPCs and ECs. Five datasets: GSE25979, GSE20283, GSE46328, GSE2040, and GSE54969 were selected for further processing (Table 1). Only samples of interest were included, excluding cells subjected to any treatment. Three studies used Affimetrix array and two used Illumina platform. Thirteen samples of early EPCs and twelve samples of ECs were included. Individual datasets were analyzed with G2R online tool (16).

**TABLE 1:** Gene expression omnibus (GEO) datasets used for this study.

GEO accession number	Platform	Total genes	EPCs/ ECs
GSE25979	Affymetrix Human Exon 1.0 ST Array	22,011	3/4
GSE20283	Illumina HumanWG-6 v3.0 expression BeadChip	21,815	3/1
GSE46328	Illumina HumanHT-12 V3.0 expression BeadChip	48,803	2/2
GSE2040	Affymetrix Human Genome U95 version 2 Array	12,554	3/3
GSE54969	Affymetrix Human Gene 1.0 ST Array	33,298	2/2

*EPCs, Circulating endothelial progenitor cells; ECs, Endothelial cells.*

### Assessment of differentially expressed genes

Meta-analysis of microarray was performed using the online tool ExpressAnalyst (17). Unprocessed data with array intensities were used as input and variance stabilizing normalization in combination with quantile normalization was performed (18). Study batch effect was adjusted using ComBat to compare information from different platforms (19). Differential expression analysis was done by Limma method, with an absolute log_2_FC > 1 (20). The cut-off *p*-values were adjusted using the Benjamini–Hochberg’s False Discovery Rate (FDR) < 0.05 (21). The combined *p*-value by Fisher exact method was computed for the intersection of datasets (22).

### Functional enrichment analysis of differentially expressed genes

Functional enrichment analysis of DEGs [GO and Kyoto Encyclopedia of Genes and Genomes (KEGG)] was carried out using KOBAS v3.0 (23); Fisher’s exact test was used to calculate *p*-values. The pathways with FDR ≤ 0.05 were defined as the significantly enriched pathways. DEGs in at least four databases were intersected with genes in principal pathways to select biologically significant genes (key genes).

### Protein-protein interaction network construction

DEGs that were significantly dysregulated in four databases were included in the PPI. The online search tool STRING (24) was used to construct the network, setting a combined score of ≥ 0.7. Cytoscape v3.7.2 was used to visualize the PPI network, and significant enriched interactions were selected by CytoHubba using maximum clique centrality score (MCC) (25).

### Micro ribonucleic acid enrichment analysis

We explored the microRNAs associated to our set of DEGs in TargetScan (26) and miRTarBase (27) databases, setting a threshold of minimum number of miRNA-target interactions > 2 and FDR < 0.05. The network was constructed in Cytoscape and intersected with the set of DEGs in at least four datasets. The key nodes were extracted by CytoHubba.

## Results

### Identification of differentially expressed genes in endothelial progenitor cells vs. mature endothelial cells

Preprocessed and normalized data were downloaded from the National Center for Biotechnology Information GEO website. Description of each dataset is available on Table 1. Two datasets were processed with Illumina platform and three datasets with Affimetrix. A total of 13 EPCs and 12 ECs samples were included for further analysis.

**FIGURE 1:**
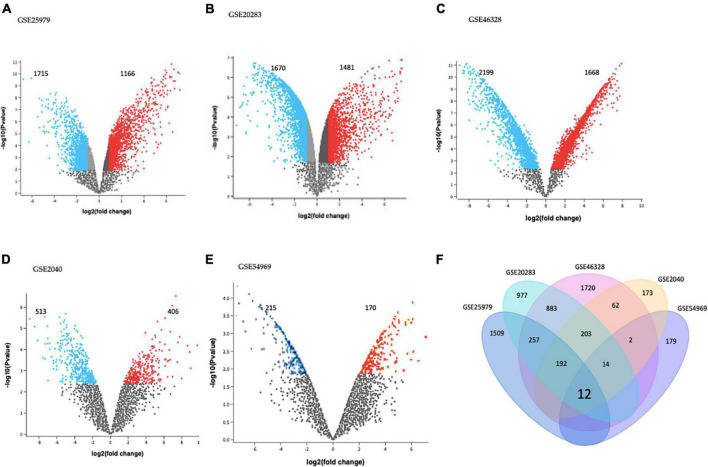
Differentially expressed genes (DEGs) on **(A)** GSE25979, **(B)** GSE20283, **(C)** GSE46328, **(D)** GSE2040, and **(E)** GSE54969 datasets. GEO2R online tool was used to identify DEGs for each dataset with | log_2_FC| > 1.0 and adjusted *p*-value < 0.05; red dots and blue dots represents the significantly down-regulated and up-regulated DEGs on EPCs, respectively. **(F)** Common DEGs before meta-analysis, by simple crossing each dataset, visualized through a Venn diagram. Twelve genes were dysregulated with the intersection of the five datasets.

GEO2R tool analysis for DEGs between EPCs and ECs on each dataset is presented using volcano plots (Figures 1A–E). Significantly dysregulated genes were defined as absolute log_2_FC > 1.0 and adjusted *p*-value < 0.05. There were 2,881 DEGs in GSE25979 (1,715 down and 1,166 up-regulated); 3,151 DEGs in GSE20283 (1,670 down and 1,481 up-regulated); 3,867 DEGs in GSE46328 (2,199 down and 1,668 up-regulated); 919 DEGs in GSE2040 (513 down and 406 up-regulated); and 385 DEGs in GSE54969 (215 down and 170 up-regulated).

Intersection of each dataset is shown in Figure 1F as a Venn diagram. Almost 63% of all DEGs were dysregulated in individual datasets. Only twelve genes were commonly dysregulated in all datasets.

Meta-analysis of microarrays was performed using the online tool ExpressAnalyst. Differential expression analysis was done by Limma method. The cut off *p*-values were adjusted using the Benjamini–Hochberg’s FDR < 0.05.

**FIGURE 2:**
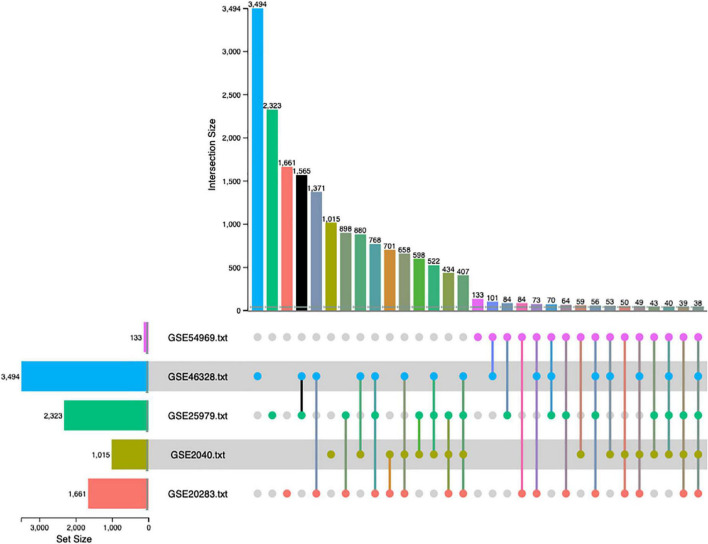
Up-set plot detailing the number of common elements among microarray datasets. Horizontal bars indicate the number of significant differentially expressed genes (DEGs) in each study. The vertical bars indicate the common elements in the sets, indicated with dots under each bar. The single points represent the number of unique elements in each group. There were 38 genes commonly dysregulated in the five datasets.

The combined *p*-value for the intersection of datasets was computed by Fisher exact method and DEGs surviving the analysis were plotted in a cord diagram. There were 38 common DEGs in the meta-analysis, 10 up-regulated and 28 down-regulated genes in EPCs (Figure 2).

### Top differentially expressed genes in the meta-analysis and functional annotation

**FIGURE 3:**
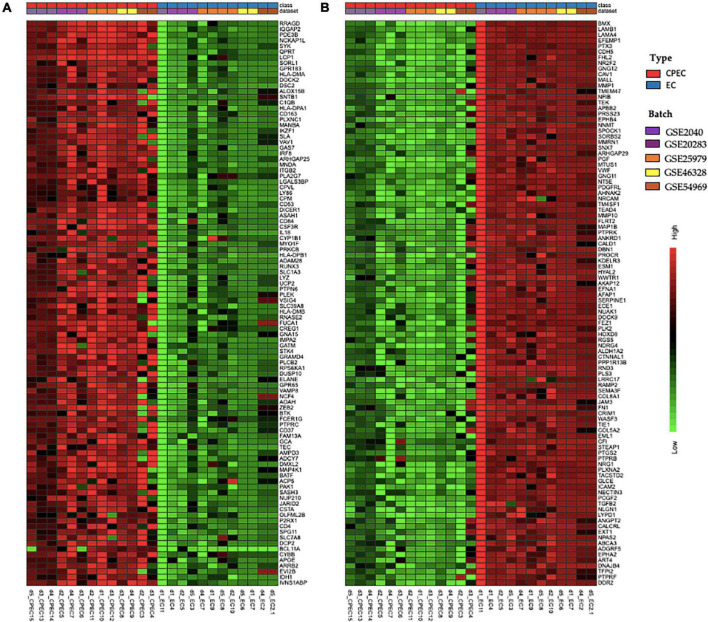
Hierarchical clustering of top 100 up-regulated DEGs **(A)** and top 100 down-regulated DEGs in EPCs **(B)**. Green and red color represent z-score expression levels, light green is the lowest and dark red is the highest value. Samples were grouped by type (EPCs and ECs) and batch. Genes were clustered by Ward method.

The meta-analysis identified 3,267 DEGs (Supplementary Table 1), 1,539 up-regulated and 1,728 down-regulated in EPCs. Figure 3 shows the top 100 up-regulated (panel A) and the top 100 down-regulated genes (panel B), with a uniform expression level among cell type.

**FIGURE 4:**
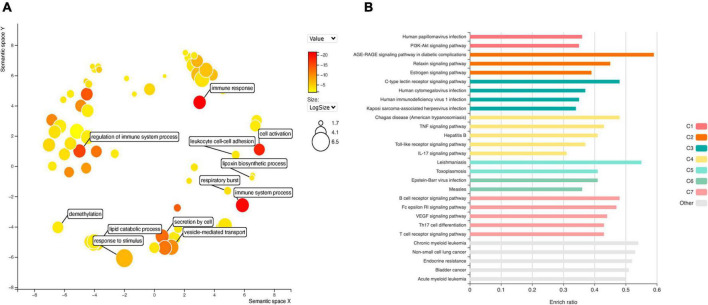
Gene ontology (GO) analysis **(A)** for DEGs in EPCs. Color intensity represents adjusted *p*-value, circle size the number or genes. The main biological processes are depicted in the graph. Functional enrichment of KEGG terms **(B)**. Color represents clusters of DEGs and bar length the enrichment ratio.

GO analysis of DEGs (Figure 4A) shows significant enrichment for terms related to cell activation (GO: 0001775), regulation of immune system process (GO: 0002682), de-methylation (GO: 0070988). While analysis of KEGG terms (Figure 4B) showed significant enrichment of “PI3K-Akt signaling pathway,” “AGE-RAGE signaling pathway in diabetic complications” and “TNF signaling pathway,” among the principal functional pathways (Supplementary Table 2).

Diabetes is one of the most important risk factors for stent restenosis; and advanced glycation end (AGE) products have a paramount importance in diabetes induced cardiovascular complications. Moreover, EPCs mobilization and homing are hampered in diabetic patients. Thus, we intersect top DEGs expressed in at least four datasets with genes overrepresented in the principal KEGG pathways, including “AGE-RAGE signaling pathway in diabetic complications”. We selected two up-regulated genes: IL1B and STAT5A; and two down-regulated: IL6 and MAPK11.

### Protein-protein interaction network analysis

**FIGURE 5:**
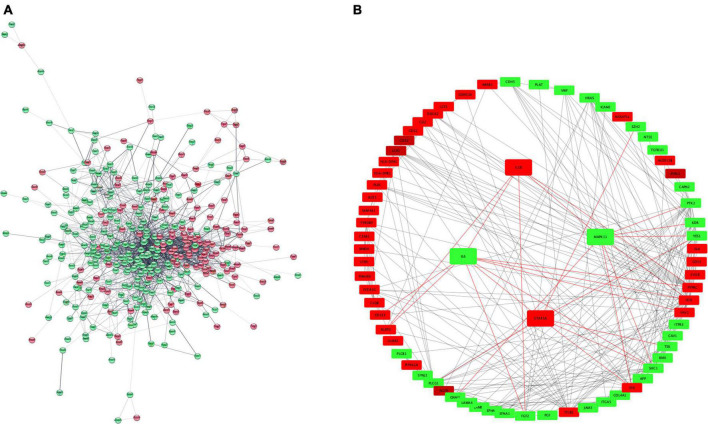
PPI network representation of 407 DEGs **(A)**. The network was constructed by STRING and visualized in Cytoscape. Hub proteins network **(B)** according to CytoHubba MCC coefficient. Red and green color represent upregulated and downregulated proteins. PPI, protein-protein interaction; MCC, maximum clique centrality. Red color represents up-regulated genes and green color down-regulated.

We conducted a PPI network analysis to explore the most significant clusters for 407 DEG. The STRING database version 11.5 was used to obtain significant interactions and resulting network was visualized in Cytoscape (Figure 5A). The MCC (maximum clique centrality) method from the CytoHubba app in Cytoscape was used to screen for key proteins (Figure 5B). There were 157 nodes, 234 edges and PPI enrichment *p*-value < 1.0e–16. The most connected hubs were HCK and VAV1 among up-regulated genes; and SHC1 and PLCG1 among down-regulated genes. The four selected genes (IL1B, STAT5A, IL6, and MAPK11) were also retained in this analysis.

### Micro ribonucleic acid enrichment analysis

**FIGURE 6:**
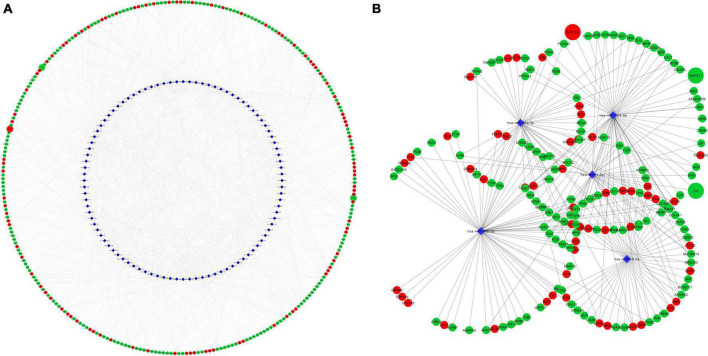
Network representation of microRNA-mRNA target interactions. **(A)** Shows interactions between 96 MicroRNA and 285 DEG in EPCs. Hub microRNAs were selected according to CytoHubba MCC coefficient **(B)**. IL6, STAT5A and MAPK11 are highlighted in larger size. Red color rep-resents up-regulated genes and green color down-regulated. Circle represents mRNA and blue diamond’s represents microRNA. MCC, maximum clique centrality.

MicroRNAs are key epigenetic regulators that modulate cell fate by means of post-transcriptional repression of gene expression. We explored microRNAs associated to our set of DEGs in TargetScan and miRTarBase databases. There were 96 microRNAs targeting 2,605 DE mRNAs, with 12,915 interactions. Intersection with the top dysregulated genes is shown in Figure 6A, with 96 microRNAs interacting with 285 DE mRNAs. Five hub microRNAs (hsa-miR-1, hsa-miR-16, hsa-miR-26b, hsa-miR-92, and hsa-miR-124) were selected (Figure 6B), targeting 175 mRNAs with 1,463 interactions. Among targeted DEGs there were STAT5A, IL6 and MAPK11. IL-6 gene was regulated by seven microRNAs, STAT5A was regulated by three microRNAs and MAPK11 only by one microRNA. MicroRNA hsa-miR-124 was a common regulator of STAT5A, IL6, and MAPK11.

## Discussion

Microarray meta-analysis is an useful strategy to reveal new associations between genes and pathological states, it is the first step of a pipeline for the discovery of new drugs or biomarkers (28). The approach used allowed us to compare a large amount of transcripts from 25 samples in two different platforms (Illumina and Affymetrix) (29).

Meta-analysis was superior than the individual dataset analysis to detect significant DEGs. Simple merging of datasets detected twelve common DEGs to all datasets, while meta-analysis detected 38. Data normalization and batch effect correction allowed comparison among multiple samples increasing the sensibility of the detection protocol (30).

EPCs are pluripotent stem cells derived from bone marrow, with the ability to home to sites of endothelial denudation (31). Considering that EPCs are extremely rare in circulating blood, *ex vivo* expansion culture systems are used to select rare EPCs using whole blood peripheral mononuclear cells (PBMCs). PBMCs are platted in fibronectin-coated dishes and exposed to growing factors (32). Early EPCs appear within 4–7 days of culture, show a limited proliferating potential for long term culture, and disappear after 2 weeks in *in vitro* conditions. They express both endothelial and monocytic markers, have a low expression of endothelial nitric oxide synthase (eNOS) and VEGFR-2 and release proangiogenic growth factors, as confirmed by transcriptomic data. Late EPCs develop from 2 to 3 weeks after plating and show a cobblestone appearance like mature ECs, expressing only endothelial markers. They show a long life span and rapidly replicate from several cells to a colony becoming a monolayer (33).

There is still a significant controversy regarding the origin of EPCs, their role and function once they migrate into the vessel. These CD34 + CD133 + early EPCs are intensively recruited after vascular injury but their proliferative potential and capacity to form mature ECs is limited. They appear to support vascular repair indirectly *via* paracrine secretory activities. On the other hand, late EPCs are capable of maturing into functioning endothelium. These cells have a higher proliferative potential and express CD31 + and KDR + (34). Comparison between early and late EPCs has also shown many dysregulated genes and proteins. These dissimilarities were pointed out by Kukumberg et al. by combining transcriptomic, proteomic and electron microscopy images analysis (35).

Due to the described differences, we only selected early EPCs samples from the datasets for further meta-analysis, as there were only three samples of late EPCs available.

EPCs are increased during vascular injury induced by balloon angioplasty and stenting, and its levels correlate with patient’s outcome (36). Patients with peripheral artery disease exhibit less EPCs, especially if associated with diabetes (37). Type 2 diabetes is one of the main clinical risk factors for vascular dysfunction and stent restenosis increasing the odds to 2–4-fold (38). In diabetic patients there is a diminished amount of EPCs, and an altered function of these cells due to premature differentiation, accelerated by hyperglycemia and increased oxidative stress (39).

The amplified inflammation in diabetes lead to increased bone marrow cell turnover, inhibiting the distribution of EPCs to ischemic tissues (40). There is also an insufficient release of marrow-stimulating factors, such as VEGFR and SDF-1, which results in downregulation of hypoxia-inducible factor (HIF-1) through the PI3K-AKT-eNOS pathway (41).

PI3K-AKT signaling pathway plays a central role in cellular physiology by mediating growth factor signals during critical cellular processes, such as glucose homeostasis, lipid metabolism, protein synthesis and cell proliferation and survival (42). VEGF and statins induce EPCs differentiation by stimulation the PI3K-AKT pathway (43). In diabetic mice, aerobic and resistance training increased PI3K-AKT pathways, improving the proliferation and adherence capacities of EPCs (44). In patients with diabetes, exercise improved *in vivo* endothelial repair capacity of EPCs by increased NO production and reduced superoxide anion level (45).

AGE products are heterogeneous groups of irreversible adducts produced by non-enzymatic glycation and glycoxidation of proteins, nucleic acid with reducing sugars (46). AGE interacts with RAGE to produce reactive oxygen species that activates nuclear factor kappa-B (NF-kB) and numerous proinflammatory genes of cytokines such as tumor necrosis factor-α (TNF-α), interleukins as IL6, and adhesion molecules (47). In an animal model, AGE-RAGE induced osteoblast differentiation of EPCs, mediated by p38, MAPK, and JNK signaling, promoting accelerated atherosclerosis (48). Enrichment of PI3K-AKT and AGE-RAGE signaling pathway for DEGs detected in meta-analysis reinforce the biological significance of our findings.

Hyperglycemia can induce EPC dysfunction triggering inflammation *via* SDF-1β/CXCR7–AMPK pathway resulting in secretion of IL6. Exendin-4, a glucagon-like peptide-1 analog could revert inhibitory effects of hyperglycemia, modulating inflammatory imbalance, restoring EPC viability and biological capacities (49).

IL6 was downregulated among EPC in our study, its potential interaction with several microRNAs (hsa-miR-1, hsa-miR-9, hsa-miR-107, hsa-miR-124, hsa-miR-98, hsa-miR-155, hsa-let-7c), seems an interesting approach to modulate EPC function.

STAT5A is part of the JAK-STAT pathway, it can form tetramers in addition to dimers. The cytokines that activate STAT5A mainly include IL3, IL2 as well as grown factors (EGF, EPO, GM-CSF, TPO, and PDGF). The biological functions of STAT5A include: (1) Regulation of growth and development. (2) Regulation of the immune system. (3) Regulation of tumor immunity. (4) Regulation of cell growth, differentiation, and apoptosis (50).

In EPCs isolated from type 2 diabetic patients there is an inhibition of STAT5/PPARγ transcriptional complex, leading to inactivation of Cyclin D1 and cell cycle arrest. Constitutive activation of STAT5 restore EPCs proliferation. The authors demonstrated that STAT5A is crucial for gene targeting and EPCs fate and the mechanisms of EPCs dysfunction in diabetic patients (51).

MicroRNA enrichment analysis detected hsa-miR-221, hsa-miR-222, and hsa-miR-124 targeting STAT5A. In breast cancer cells, repression of hsa-miR-221 and hsa-miR-222 were associated to overexpression of STAT5A conferring a more aggressive tumor phenotype (52). In hyperglycemic induced damage of mesangial cells, hsa-miR-222 transfer *via* extracellular vesicle, derived in STAT5A repression, protecting cells from hyperglycemic injury (53).

The p38 MAPK family is composed of α, β (MAPK11), γ and δ, isoforms which are encoded by separate genes. These kinases transduce extracellular signals and coordinate the cellular responses needed for adaptation and survival. However, in cardiovascular and other disease states, these same systems can trigger maladaptive responses that aggravate, rather than alleviate, the disease (54). Its activation in ECs leads to actin remodeling, angiogenesis, DNA damage response and thereby has major impact on cardiovascular homeostasis, and on cancer progression (55).

Seeger et al. showed that p38 MAPK plays a pivotal role in the signal transduction pathways regulating the number of EPCs. EPCs from patients with coronary artery disease had significantly higher basal p38-phosphorylation levels compared with healthy subjects. Additionally, TNF-α and glucose induced a dose- and time-dependent activation of the p38 MAP kinase in healthy EPCs. Treatment with SB203580, an inhibitor of p38-kinase could reverse impaired capacity for neovascularization and augmented EPCs number (56).

Other study demonstrated the deleterious effect of hyperglycemia on EPCs *via* p38 MAPK phosphorylation. The exposure of cultured EPCs to high glucose significantly accelerated the rate of senescence compared with that in osmolar control during culture. The phosphorylation of p38 MAPK in EPCs was increased by glucose compared with control in a dose-dependent manner. Hyperglycemia-induced EPC senescence was significantly inhibited by the addition of an inhibitor of the p38 MAPK, SB203580 (57).

Hsa-miR-124 has been shown to target p38 MAPK *in silico* as well as in experimental models (58). The expression levels of hsa-miR-124 and p38 MAPK showed and inverse correlation in patients with coronary artery disease with over expression of p38 MAPK and repression of hsa-miR-124. Furthermore hsa-miR-124 transduction in macrophages inhibited apoptosis *via* targeting p38 MAPK signaling pathway in atherosclerosis development (59).

Chang et al. deciphered the microRNA profile of EPCs (early and late) and ECs by small RNA sequencing. Of note, among top five selected microRNAs, only hsa-miR-124 was overexpressed in early EPCs, with null expression in late EPCs and ECs (60).

## Conclusion

The present study guided to the identification of a set of mRNAs dysregulated during the differentiation of circulating early EPCs into mature ECs, specially related to AGE/RAGE signaling pathway in diabetic complications. Furthermore, a group of microRNAs potentially associated with key differentially expressed mRNAs was highlighted. We hypothesize that overexpression of hsa-miR-124 might repress IL6 and p38β/MAPK11, allowing EPCs differentiation into mature ECs.

This group of genes could serve as biomarkers of EPCs differentiation during coronary stenting as well as potential therapeutic targets to improve stent re-endothelization, especially in diabetic patients. However, further studies are needed to validate and explore the impact of the proposed genes on EPCs.

## Data availability statement

The datasets presented in this study can be found in online repositories. The names of the repository/repositories and accession number(s) can be found in the article/Supplementary material.

## Author contributions

AA and LS: conceptualization. AA: methodology, formal analysis, investigation, data curation, writing—original draft preparation, and visualization. LS: resources, writing—review and editing, supervision, project administration, and funding acquisition. Both authors have read and agreed to the published version of the manuscript.
